# *Terminalia bellirica* and *Andrographis paniculata* dietary supplementation in mitigating heat stress-induced behavioral, metabolic and genetic alterations in broiler chickens

**DOI:** 10.1186/s12917-024-04233-2

**Published:** 2024-09-03

**Authors:** Rabie H. Fayed, Sara E. Ali, Aya M. Yassin, K. Madian, Basma M. Bawish

**Affiliations:** 1 Department of Veterinary Hygiene and Management, Faculty of Veterinary Medicine, Cairo University, Giza, 12211 Egypt; 2 Department of Physiology, Faculty of Veterinary Medicine, Cairo University, Giza, 12211 Egypt; 3 Department of Biochemistry and Molecular Biology, Faculty of Veterinary Medicine, Cairo University, Giza, 12211 Egypt; 4 Department of Poultry Diseases, Faculty of Veterinary Medicine, Cairo University, Giza, 12211 Egypt

**Keywords:** *Andrographis paniculata*, *HSP70*, Oxidative stress, *Terminalia bellirica*, *Welfare*

## Abstract

**Background:**

Heat stress (HS) is one of the most significant environmental stressors on poultry production and welfare worldwide. Identification of innovative and effective solutions is necessary. This study evaluated the effects of phytogenic feed additives (PHY) containing *Terminalia bellirica and Andrographis paniculata* on behavioral patterns, hematological and biochemical parameters, Oxidative stress biomarkers, and *HSP70*,* I-FABP2*,* IL10*,* TLR4*, and mTOR genes expression in different organs of broiler chickens under chronic HS conditions. A total of 208 one-day-old Avian-480 broiler chicks were randomly allocated into four treatments (4 replicate/treatment, 52 birds/treatment): Thermoneutral control treatment (TN, fed basal diet); Thermoneutral treatment (TN, fed basal diet + 1 kg/ton feed PHY); Heat stress treatment (HS, fed basal diet); Heat stress treatment (HS, fed basal diet + 1 kg/ton feed PHY).

**Results:**

The findings of the study indicate that HS led to a decrease in feeding, foraging, walking, and comfort behavior while increasing drinking and resting behavior, also HS increased red, and white blood cells (RBCs and WBCs) counts, and the heterophile/ lymphocyte (H/L) ratio (*P < 0.05);* while both mean corpuscular volume (MCV), and mean corpuscular hemoglobin (MCH) were decreased (*P* < 0.05). In addition, HS negatively impacted lipid, protein, and glucose levels, liver and kidney function tests, and oxidative biomarkers by increasing malondialdehyde (MDA) levels and decreasing reduced glutathion (GSH) activity (*P < 0.05*). Heat stress (HS) caused the upregulation in *HSP70*, duodenal *TLR4* gene expression, and the downregulation of *I-FABP2*, *IL10*, *mTOR* in all investigated tissues, and hepatic *TLR4* (*P < 0.05*) compared with the TN treatment. Phytogenic feed additives (PHY) effectively mitigated heat stress’s negative impacts on broilers via an improvement of broilers’ behavior, hematological, biochemical, and oxidative stress biomarkers with a marked decrease in *HSP70* expression levels while all tissues showed increased *I-FABP2*, *IL10*, *TLR4*, and *mTOR* (except liver) levels (*P < 0.05*).

**Conclusion:**

Phytogenic feed additives (PHY) containing *Terminalia bellirica and Andrographis paniculata* have ameliorated the HS-induced oxidative stress and improved the immunity as well as the gut health and welfare of broiler chickens.

## Introduction

Heat stress (HS) is one of the most significant environmental stressors in livestock and poultry production around the world [1], resulting in a significant economic loss that is expected to surge in the next years as global temperatures [2, 3]. The HS occurs when an animal’s body generates more heat than it dissipates to its immediate environment [4]. Broilers are sensitive to HS conditions for many reasons such as the fast growth rate, high metabolic rate, and lack of sweat glands in their skin [5, 6]. The HS poses a significant threat to the poultry industry, and this concern will intensify as climate change continues to elevate environmental temperatures [2, 3]. Under HS circumstances, the physiological blood parameters of broilers are changed, resulting in changes in behavioral patterns and impairing their welfare [6, 7]. The detrimental effects of HS on avian physiology and behavior are mainly mediated by the development of oxidative stress and redox imbalance [8, 9]. Previous studies have shown that long-term HS can disrupt the body’s redox balance, resulting in the formation of reactive oxygen species (ROS), and oxidative damage that harms metabolism in chickens [10, 11]. Elevated ambient temperature causes oxidative stress in broiler liver tissues, exacerbating the disruption of lipid metabolism [12]. Free radicals rise in broilers under HS, while antioxidant enzyme activity and the capacity to scavenge free radicals fall [13]. Additionally, Ghanima et al. [11]; Ma et al. [14] showed that exposure to HS suppresses the innate immune response and induces immune disorders via altering the organs’ immune functions.

In an adaptive response to HS in poultry, the sympathetic adreno-medullary (SAM) pathway is activated. This leads to the release of catecholamines from the adrenal medulla, including epinephrine and norepinephrine. Additionally, the hypothalamic-pituitary-adrenal (HPA) axis is activated, which causes the adrenal cortex to secrete glucocorticoids (GC), such as cortisol and corticosterone [15–18]. The high corticosterone levels can in turn weaken the intestinal barrier. In chickens exposed to HS, damage to the intestinal barrier can cause intestinal inflammation. This has been shown to reduce the life span of intestinal cells, cause crypt hyperplasia and villous atrophy in ducks [19], and raise the concentration of plasma endotoxins in chickens [20].

The body’s HS response is exacerbated when the balance of pro- and anti-inflammatory factors is disrupted in the digestive tract or throughout the body [21]. Defense mechanisms that shield cells from heat-induced stress include the expression of early stress response genes that code for heat shock protein (HSP); genes related to the immune system, such as cytokines and toll-like receptors (TLRs); and antioxidant enzymes, such as superoxide dismutase (SOD) and catalase (CAT) [22].

Mitigation strategies are crucial for alleviating the deleterious effects of environmental stressors and climate change on broiler behavioral response, immune functions, and antioxidant capacity, particularly under HS conditions. Several mitigating techniques have previously been employed, including feed additives such as trace elements [3, 23] vitamins [24, 25], probiotics [26], and herbal products [27, 28]. It has been demonstrated that using herbs and their extracts in poultry diets is typically useful in preserving their health, behavior, and welfare, as well as oxidative stability and metabolism [29]. *Terminalia bellirica* (Gaertn.) Roxb. (Family Combretaceae), commonly known as ‘Belleric myrobalan,’ is a huge deciduous tree native to the Indian subcontinent, Nepal, Sri Lanka, and Southeast Asia [30]. *T. bellirica* is linked to the diverse pharmacological effects exhibited by its various bioactive secondary metabolites, which include alkaloids, flavones, lignans, tannins, phenols, terpenoids, glycosides, and saponins [31]. Modern studies suggest that the various chemicals in *T. bellirica* may be responsible for its acclaimed health benefits, such as antipyretic, antioxidant, anti-inflammatory, and immunomodulatory Activity [32, 33]. *T. bellirica* components were proven to boost macrophage activities and immune response in terms of free radical scavenging and reactive oxygen species neutralization [34].

*Andrographis paniculata* is a widely grown herb in East and Southeast Asia that belongs to the Acanthaceae family [35]. It has been identified as the “King of Bitter” because of its capacity to thrive in diverse soil types and shady situations [36]. *A. paniculata* Secondary metabolites including terpenoids, saponins, phenols, alkaloids, glycoside, and flavonoids have several pharmacological activities [37]. *A. paniculata* has anti-inflammatory, antibacterial, antipyretic, and immunostimulant activities [38]. It has been proven that *A. paniculata* considerably increases the antioxidant, and immunological function in broiler chicken [39, 40]. **Gao et al.**, **(2022)** [41] reported that the polyherbal supplement including *A. paniculata* increased blood IgG levels in broiler chicks, suggesting that it protects the immune system and reduces inflammatory cytokine production.

The possible interactions between the various combinations of *T. bellirica* and *A. paniculata* have not been thoroughly investigated in broiler chickens subjected to HS environment. Therefore, this experiment aims to determine the effect of the dietary use of *T. bellirica* and *A. paniculata* combination on the behavioral patterns, haematological, biochemical, Oxidative stress biomarkers, and *HSP70*,* mTOR*,* TLR4*,* IL10*, and I-FABP2 genes expression in broiler chickens under chronic HS conditions.

## Materials and methods

### Ethical approval

The husbandry and the following procedures were approved by the Institutional Animal Care and Use Committee (IACUC), Faculty of Veterinary Medicine, Cairo University, Egypt ((Ethical reference No: Vet CU 09092023779) and in accordance to ARRIVE guidelines.

### Experimental design

#### Housing and diets

A total of 208 one-day-old Avian-480 broiler chicks were obtained from a commercial hatchery, which were individually weighed and placed in 16-floor pens (1 × 1.3 m^2^, 13 chicks/pen). The pens were located in 2 environmentally controlled rooms (8 pens each, 104 birds/room) at the Poultry Research Unit, Department of Veterinary Hygiene and Management, Faculty of Veterinary Medicine, Cairo University, Egypt. Each pen is covered with 8 cm fresh wood shaving and equipped with separate feeders and drinkers.

The experiment was conducted in a complete randomized design with two experimental diets within each room for 35 days: A three-phase corn-soybean-based diet formulated to fulfill Avian-480 broilers’ nutritional needs [42] (Table 1), and a diet fortified with the (1 kg/ton feed) Phytogenic feed additive (PHY) “Herb-ALL^™^ COOL” preparation, (Life Circle Nutrition AG, Häm¬merli 2d, 8855, Wangen SZ, Switzerland), according to the manufacturer’s recommendation. The “Herb-ALL^™^ COOL” was added to the diet during the three growing phases: starter (d0–14), grower (d15–28), and finisher (d29–35) as recommended by the manufacturer. The birds were fed a mash diet for one to thirty-five days to ensure adequate mixing. *Terminalia bellirica* and *Andrographis paniculata* are two of the pre-standardized, tested herbs in the polyherbal preparation “Herb-ALL^™^ COOL”. The herbal composition (as provided by the manufacturer) is as follows: polyphenols: 3.32 g GAE /100 g (GAE: gallic acid equivalents), 25.7% (%W/W) water-soluble extract value, 13.5% crude fiber, 6.5% crude protein, 2.5% crude fat, 8.5% crude ash, 0.02% sodium, 0.2% lysine, and 0.1% methionine, 8.9% humidity. Feed and water were available *ad libitum*.

**Table 1:** Ingredients and nutrient composition of the basal diets for each growing period

**Ingredients%**	Starter (0 to 14 days)	grower (15 to 28 days)	finisher (29 to 35 days)
**Yellow corn**	57.94	61.67	66.65
**Soybean meal 46%**	37.0	33.5	28.5
**Soy oil**	1.40	1.50	2.00
**Limestone**	1.40	1.40	1.30
**Monocalcium phosphate**	0.7	0.55	0.5
**Sodium bicarbonate**	0.15	0.15	0.15
**Salt**	0.2	0.2	0.2
**DL. Methionine**	0.35	0.3	0
**L. Lysine HCl 78.5%**	0.31	0.23	0.23
**L- Valine**	0.01	0	0
**L. Therionine**	0.13	0.1	0.07
**Choline chloride 60%**	0.07	0.07	0.07
**Vitamin mineral premix** ^**1**^	0.21	0.2	0.2
**Mycotoxin binder** ^**2**^	0.10	0.10	0.10
**Total** ^**3**^	100	100	100
**Chemical analysis**:			
**Metabolizable Energy (Kcal/kg)**	3100	3150	3240
**Crude Protein (%)**	23.00	21.5	19.5
**Crude Fiber**	2.3	2.2	2.2
**Crude Fat (%)**	4.5	4.7	5.5
**Calcium (%)**	1.00	0.95	0.9
**Available phosphorus (%)**	0.48	0.435	0.395

^1^ Vitamin and mineral mixture contained: 13,000,000 IU vitamin A; 80,000 mg vitamin E; 6,000,000 IU vitamin D3; 4000 mg vitamin K; 5000 mg vitamin B1; 9000 mg vitamin B2; 5000 mg vitamin B6; 35 mg vitamin B12; 20,000 mg pantothenic acid; 2000 mg Folic acid; 70,000 mg Nicotinic acid; 250 mg Biotin; 100,000 mg Zinc oxide; 400,000 mg Manganese oxide; 1000 calcium Iodide; 15,000 mg Copper sulphate; 350 mg Selenium selenite; 50,000 mg ferrous sulphate

^2^ Mycotoxin binder: Rovi-yeast

^3^ Phytase (PHYTASE HT8 5 K) = 0.02% and Energy enzyme (Axtra^®^ XB 201) Xylanase and Beta glucanase = 0.01%

#### Heat-challenged environment and bird allocation

The ambient temperature in room 1 (thermoneutral (TN) treatments) gradually decreased from 33◦C at 1d to 24◦C at 21 d (0.5◦C/d) and maintained for the rest of the experiment. In the 2nd room (heat stressed (HS) treatments, the temperature was maintained at 33 °C in the 1st week and 30 °C in the 2nd week, then the temperature was kept at 32◦C from the 3rd week for 10 h (8 am–6 pm) daily and was reduced to 24 °C each night until the end of the trial [43]. This creates four experimental treatments (4 replicate/treatment, 52 birds/treatment): Thermoneutral control treatment (TN, fed basal diet); Thermoneutral treatment (TN, fed basal diet + 1 kg/ton feed PHY); Heat stress treatment (HS, fed basal diet); Heat stress treatment (HS, fed basal diet + 1 kg/ton feed PHY). The relative humidity (RH) in both rooms ranged between 55 and 60%. The lighting program was 24 h. light for the first three days, then 23 L:1D for the rest of the trial. The vaccination program was Newcastle disease virus (NDV) Hitchner B1 vaccination on the 6th day, NDV-Lasota vaccination and Avian Influenza (H5N1) on the 10th day, and Infectious Bursal Disease (IBD) on the 14th day.

#### Behavioral observation

The behavioral observation was started from 7 to 35 days old and recorded using a video camera. According to [44], the scanning technique was used to observe bird behavior directly throughout the investigation. All birds in each replicate were observed two days/week, each replicate was observed 10 min daily, and the number of birds performing each behavior pattern was recorded once every 1 min (i.e. each replicate scored 10 times/day). Then the percentage of chicks performing the following behaviors was recorded: feeding, drinking, standing/walking, resting, floor pecking, wing stretching, preening, head-scratching, dust bathing, and wing flapping [45].

#### Blood sampling

At the end of the trial (day 35 ), The birds were fasted overnight, then slaughtered by severing the jugular vein using a sharp knife, and then the sacrificed birds were de-feathered and eviscerated. Three blood samples were randomly taken from 3 birds/ replicate in each treatment, the first was collected on an EDTA anticoagulant tube for hematological parameters estimation, the second on a heparinized anticoagulant tube for GSH activity and centrifuged at 5000 rpm/20 min for obtaining plasma sample for MDA and TAC determination, the third was on a gel separator tube centrifuged at 5000 rpm/20 min to obtain serum samples for metabolic parameters, liver, and kidney function tests.

#### Tissue sampling

60 mg of the liver, duodenum, and jejunum specimens were cut, gathered on liquid nitrogen, and kept at -80 °C to extract RNA.

#### Hematological parameters

The hematocrit (HTC) percentage was estimated using the method described by [46]. Hemoglobin (Hb) concentration was determined using commercial kits (Biodiagnostic Company, Egypt) following the [47] method. Erythrocytes (RBCs) were manually counted using a hemocytometer counting chamber based on the [47] method. Blood indices were calculated following the [46] method. The mean corpuscular volume (MCV) was calculated as (HTC*10)/RBCs count, the mean corpuscular hemoglobin (MCH) as (Hb*10)/RBCs count, and the mean corpuscular hemoglobin concentration (MCHC %) as (Hb/ HTC) * 100. The leukocytes (WBCs) and differential leukocytic counts were estimated following the [48] method. Heterophils and lymphocytes were identified based on their characteristics as described by [49]. The heterophil to lymphocyte (H: L) ratio was then calculated [50].

#### Metabolic parameters

Serum Metabolic parameters including lipid, protein profiles, and blood glucose levels were measured spectrophotometrically using commercial kits supplied by Spectrum Diagnostics Egyptian Company for Biotechnology; according to the manufacturer’s instructions (UV-2100 Spectrophotometer, USA) [51].

The lipid profile including total cholesterol, total glycerides, and HDL cholesterol concentrations were estimated following the methods described by [52, 53], and [54]; respectively, while LDL and VLDL cholesterol concentrations were calculated according to [55].

VLDL cholesterol concentration = Triglycerides/5.

LDL cholesterol concentration = Total cholesterol – (HDL + VLDL).

Briefly, a series of reactions are involved in the cholesterol assay; cholesterol esters are enzymatically hydrolyzed by cholesterol esterase to produce cholesterol and free fatty acids. The resultant cholesterol, in turn, oxidized by cholesterol oxidase to cholest-4-en-3-one and hydrogen peroxide. Triglycerides assessment requires the action of lipoprotein lipase (LPL) to produce glycerol which is then phosphorylated to glycerol-3-phosphate by glycerol kinase in the presence of ATP. Glycerol-3-phosphate is then oxidized by glycerol phosphate oxidase forming dihydroxy acetone phosphate and hydrogen peroxide. Hydrogen peroxide, either from cholesterol or triglycerides, reacts with phenol and 4 amino antipyrine in the presence of peroxidase forming quinoneimine dye which can be quantified at 546 nm.

For HDL cholesterol quantification, LDL and VLDL in the samples precipitate with phosphotungstate and magnesium ions. after centrifugation at 4000 rpm for 10 min, the cholesterol of HDL fraction remains in the supernatant which can be determined with the same procedures as total cholesterol.

The protein profile -including total protein, and albumin was estimated colorimetrically following the methods described by [56, 57]. On the other hand, globulin was calculated as total protein minus albumin and A/G ratio according to [58]. Briefly, Protein quantification depends on the reaction of copper with peptide bonds of proteins to form a characteristic pink-to-purple biuret complex that can be assessed at 546 nm. The measurement of albumin is based on its binding to bromocresol green (BCG) in pH 4.1, forming a blue-green colored complex measured at 623 nm.

Blood glucose level was estimated according to [54] where glucose is enzymatically oxidized by glucose oxidase to produce hydrogen peroxide which in turn reacts with phenol and 4 aminoantipyrine under the catalysis of peroxidase to produce red violet quinoneimine dye measured at 546 nm.

#### Liver and kidney function biomarkers

Liver and kidney function parameters were evaluated in serum using commercial kits provided by Spectrum, Egypt. The activities of liver enzymes, alanine aminotransferase (ALT) and aspartate aminotransferase (AST), were assessed based on the method established by [52]. Kidney function tests, including uric acid and creatinine levels, were measured according to the procedures described by [56, 57]; respectively.

#### Oxidative stress biomarkers

Reduced glutathione (GSH) activity was determined using heparinized whole blood, while the total antioxidant capacity (TAC) and malondialdehyde (MDA) levels were estimated in heparinized plasma following the instructions supplied by the manufacturer of the commercial Kits (Biodiagnostic Company, Egypt) according to [59, 60], and [61]; respectively.

Briefly; the measurement of GSH depends on the reduction of 5,5’ dithiobis (2-nitrobenzoic acid ) (DTNB) with GSH to produce a yellow compound which can be measured at 405 nm and results are expressed as mg/dl. The determination of TAC depends on the elimination of exogenously added hydrogen peroxide by the anti-oxidants in the sample. The residual hydrogen peroxide is determined colorimetrically by an enzymatic reaction which involves the conversion of 3,5 dichloro-2-hydroxy benzenesulfonate to a colored product measured at 505 nm and the results expressed as mM/L. MDA is a degradation product of lipid peroxides and was determined using the thiobarbituric acid (TBA) method. MDA condenses with TBA to form a red product with a maximum absorption peak at 534 nm and data are expressed as nmol/ml.

#### Quantitative real-time RT-PCR analysis

Total RNA was extracted from the liver, duodenum, and jejunum tissues, measuring its concentration and purity, and reverse transcribed according to [62] using total RNA purification kit **(Jena Bioscience**,** Germany**,** Cat. #PP-210 S)** and Revert Aid First Strand cDNA Synthesis Kit **(Thermo Scientific**,** USA**,** Cat. #K1622)** with following the manufacturer instruction. The fluorescence-based real-time detection method was employed to estimate each gene’s level of mRNA expression relative to β-actin (ACTB), an endogenous reference gene. The method used iQ SYBR^®^ Green Supermix **(Bio-Rad 1708880, USA)**. The primers listed in Table 2 were used to perform real-time RT-PCR for every gene. The cycling protocol involved an initial denaturation of the sample for three minutes at 95 °C, followed by 40 cycles of denaturation (15 s at 95 °C), annealing (30 s at 60 °C), and extending (30 s at 72 °C). At the end of each reaction, the melting curves were examined to determine the specificity of the PCR products. Every experiment was carried out in three triplicates with a no-template negative control (NTC) present. The expression in comparison to the control was determined by applying the Eq. 2-ΔΔCT [63].

**Table 2:** Primers sequences used for qRT-PCR analysis

Gene symbol		Amplicon size (bp)	Gene description	Accession number/REF.	Primer Sequence
**1**	**House keeping gene:**				
***β-actin (ACTB)***		**177 bp**	Beta-actin	L08165.1	F:- 5′-CCCACACCCCTGTGATGAAA-3′ R:- 5′-TAGAACTTTGGGGGCGTTCG-3′
**2**	**Heat stress responsive gene**:				
***HSP-70***		**145 bp**	Heat shock protein − 70	**Calik et al.**,** 2022 **[1]	F: 5′-CGTCAGTGCTGTGGACAAGAGTA-3′ R: 5′-CCTATCTCTGTTGGCTTCATCCT-3′
**3**	**Gut health biomarker**:				
**I-FABP-2**		**77 bp**	Intestinal fatty acid binding protein 2	**Chen et al.**,** 2015 **[140]	F:5-′AAAGATAATGGAAAAGTACTCACAGCAT-3′ R:5′-CCTTCGTACACGTAGGTCTGTATGA-3′
**4**	**Immunity related genes**:				
***IL-10***		**174 bp**	Interleukin-10	NM_001004414.4	F: 5′- TGTACCATTTGTGGCAGTGC − 3′ R: 5′- TCGTCTGGTGTTTGCAGTTG − 3′
***TLR-4***		**158 bp**	Toll-like receptor 4	NM_001030693.1	F: 5′-ATGTCCTCTTGCCATCCCAA-3′ R: 5′-TCTCCCCTTTCTGCAGAGTG-3′
**5**	**Nutrient sensing pathway**:				
***mTOR***		**213 bp**	Mechanistic Target of Rapamycin	XM_417614.6	F: 5′-CGCAGTGAAGAAACAAGGGC-3′ R: 5′-GGTGGCGTTACCTCCTTCAA-3′

### Statistical analysis

The Shapiro-Wilk test was used to verify for normality, and the Levene test was used to check for variance homogeneity. The mean and standard error were calculated for each variable. The data were analyzed by analysis of variance (ANOVA) to identify the significantly different treatments at (*P < 0.05*) by one-way ANOVA using the SPSS software statistical program (SPSS for Windows ver.27, USA). Graph Pad Prism version 6.0 was used to create the graphs.

## Results

### Behavioral observation

**Fig. 1:**
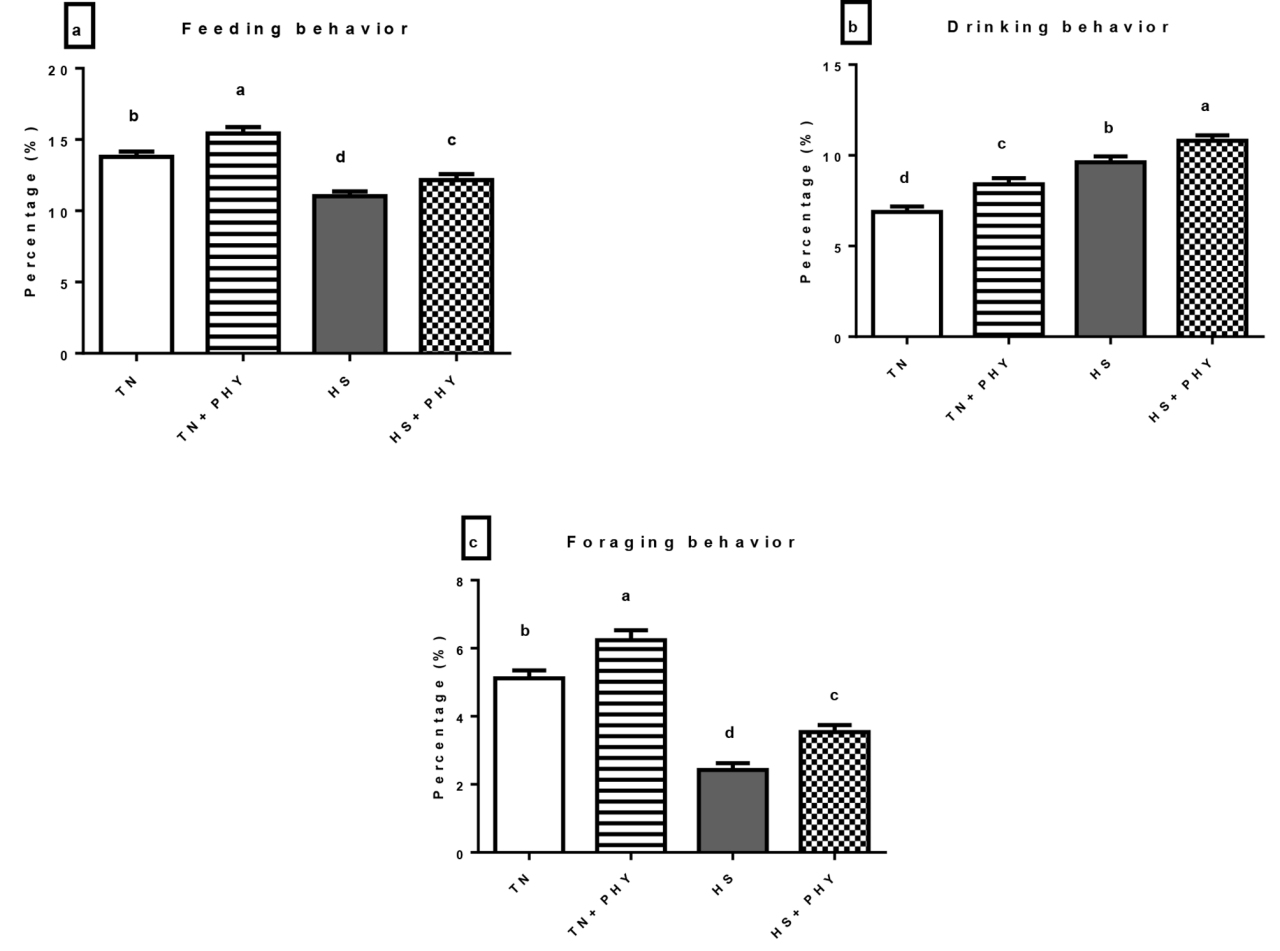
Effect of phytogenic feed additives (PHY) on **(a)** feeding, **(b)** drinking, and **(c)** foraging behavior percentage of heat-stressed broiler chickens; Data are presented as mean ± standard error. Different alphabets indicate significant differences at *P* < 0.05

**Fig. 2:**
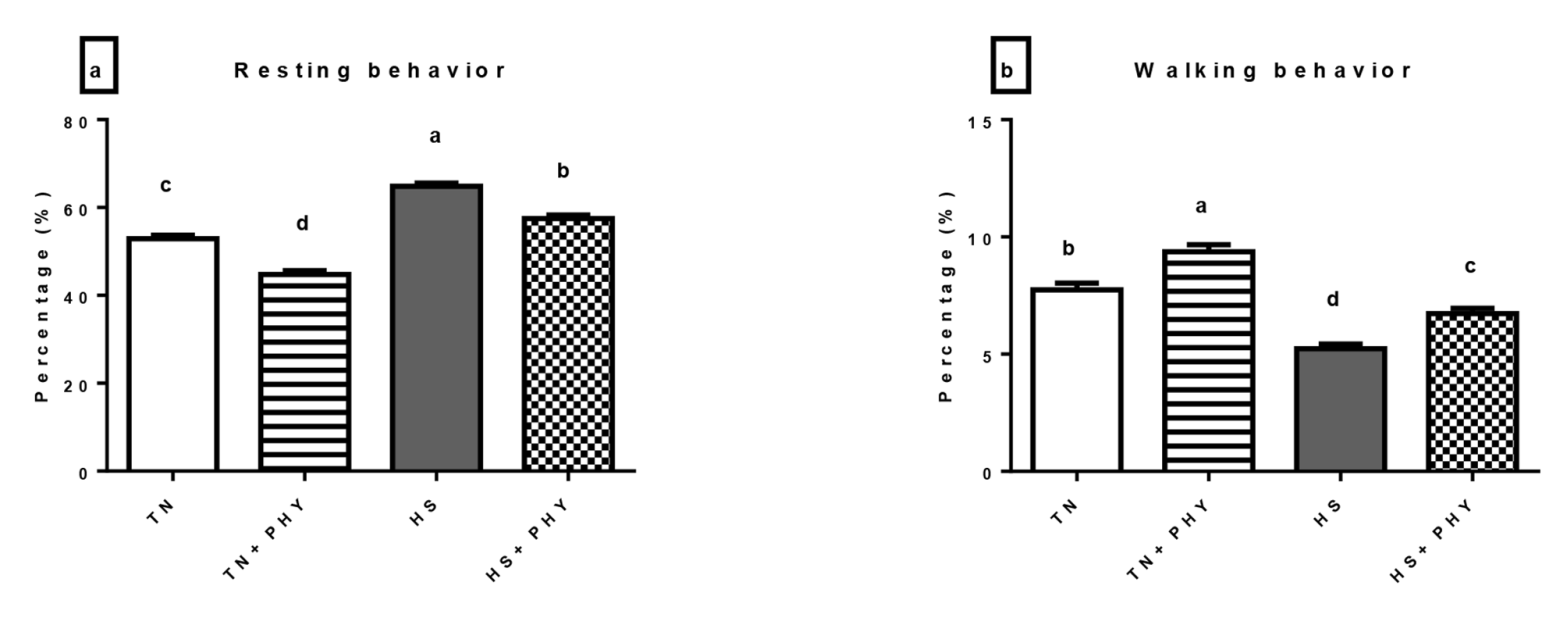
Effect of phytogenic feed additives (PHY) on **(a)** resting, and **(b)** walking behavior percentage of heat-stressed broiler chickens; Data are presented as mean ± standard error. Different alphabets indicate significant differences at *P* < 0.05

**Fig. 3:**
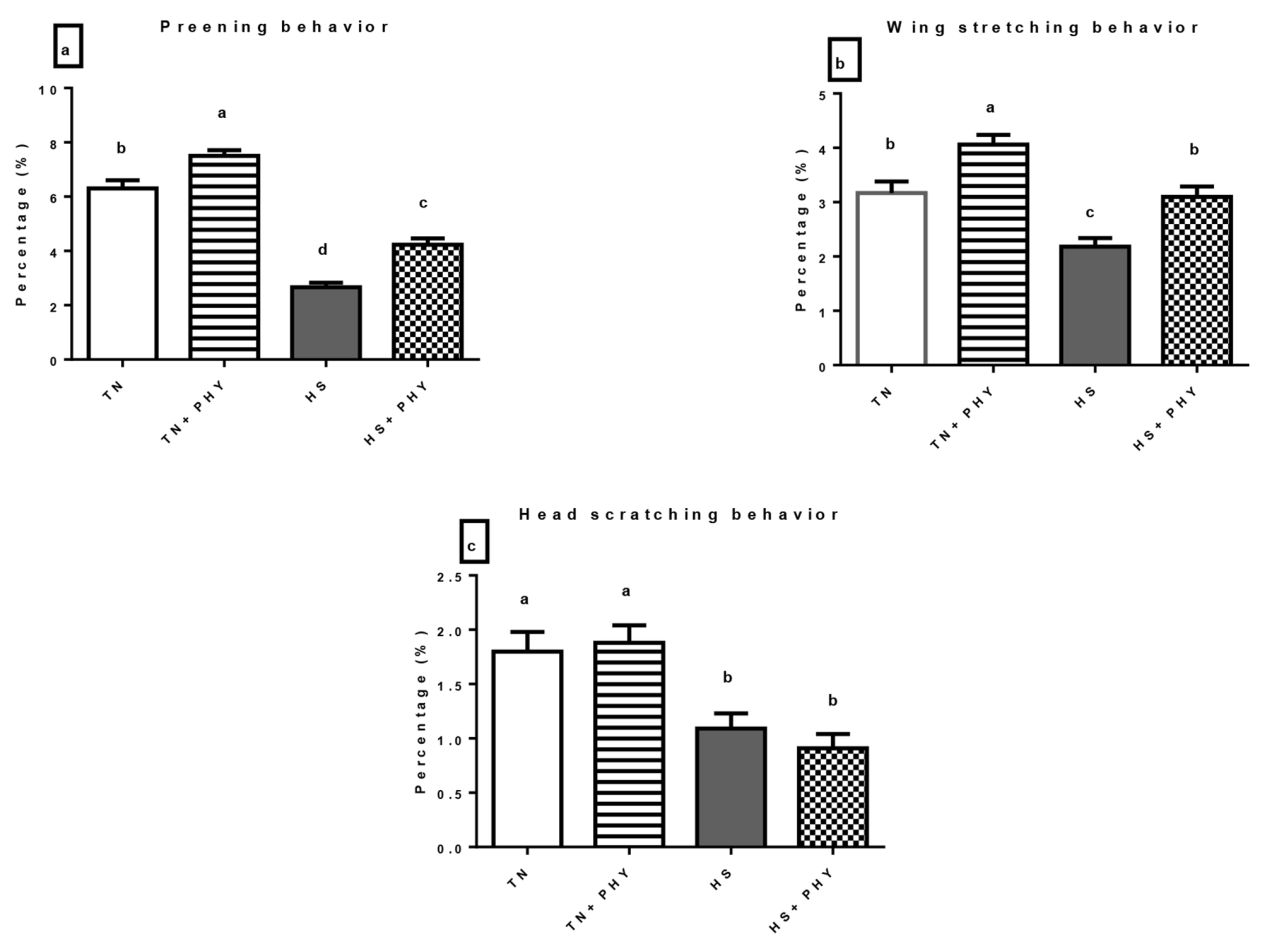
Effect of phytogenic feed additives (PHY) on **(a)** preening, **(b)** wing stretching, and **(c)** head-scratching behavior percentage of heat-stressed broiler chickens; Data are presented as mean ± standard error. Different alphabets indicate significant differences at *P* < 0.05

**Fig. 4:**
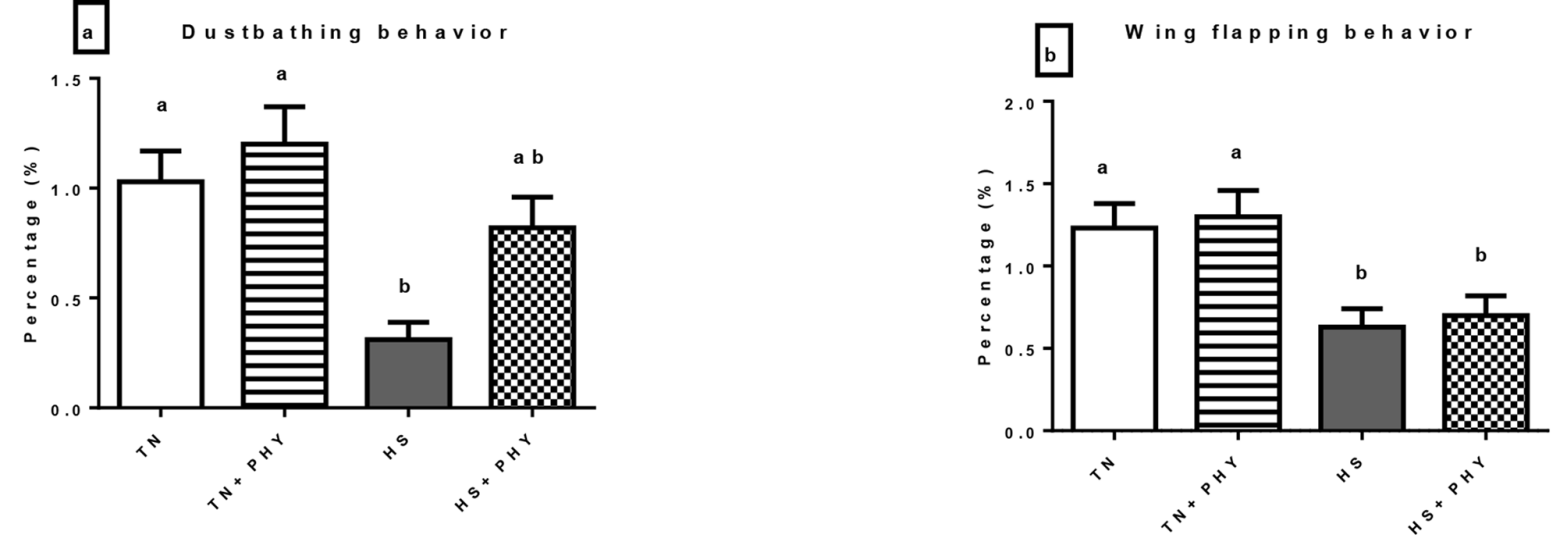
Effect of phytogenic feed additives (PHY) on (a) dust bathing, and (b) wing flapping behavior percentage of heat-stressed broiler chickens; Data are presented as mean ± standard error. Different alphabets indicate significant differences at *P* < 0.05

The data presented in Figs. (1–4) demonstrated the effect of PHY feed additives as an anti-heat stressors on the different behavioral patterns percentage in broiler chickens subjected to chronic HS. A significant decrease in the feeding behavior and an increase in the drinking behavior were noticed in the HS treatment compared with the TN (Cont.) treatment (*P < 0.05*), Moreover, there was a significant decrease in walking and foraging behavior and an increase in the resting behavior in HS treatment versus the TN treatment (*P < 0.05*). The comfort behavior (preening, wing stretching, head-scratching, dust bathing, and wing flapping was significantly decreased in the HS treatment compared with the TN treatment (*P < 0.05*) .

The PHY fortification under HS conditions in (HS + PHY) treatment increased the feeding, drinking, foraging, and walking behavior while the resting behavior was significantly reduced when compared to the HS treatment (*P < 0.05*). The comfort behavior improved significantly (*P < 0.05*) in the HS + PHY treatment as observed in preening and wing stretching behavior with no significant effect on head scratching, dust bathing, and wing flapping behavior in comparison to the HS treatment (*P > 0.05*). Additionally, the PHY feed additive under TN conditions showed an increase in the feeding, drinking, foraging, walking, preening, and wing stretching behavior while the resting behavior decreased in the TN + PHY treatment compared to the TN treatment (*P < 0.05*), while no significant effect on the other comfort behavior between them (*P > 0.05*).

### Hematological parameters

The hematological parameters presented in Table 3 show that the HS treatment experienced a significant increase in RBCs count, while both MCV and MCH decreased compared to the TN treatment (*P < 0.05*). Additionally, the WBCs count was elevated, primarily due to a rise in heterophile % and reduction in the lymphocyte % leading to a significant increase in the H/L ratio when compared with the TN treatment (*P < 0.05*). The other hematological tests in the HS treatment did not change significantly compared to the TN treatment. The treatments receiving PHY herbal fortification (TN + PHY and HS + PHY) did not display significant changes in hematological parameters when compared to the TN treatment (*P > 0.05*).

**Table 3:** Effect of phytogenic feed additives (PHY) on hematological parameters of thermoneutral, and heat-stressed broiler chickens

**Parameters**	TN	TN + PHY	HS	HS + PHY
**Hematocrit (%)**	37.8 ± 0.37 ^**a**^	38.2 ± 0.58 ^**a**^	39.4 ± 0.51 ^**a**^	38.4 ± 0.51 ^**a**^
**Hb conc (g/dl)**	7.82 ± 0.06 ^**a**^	8.16 ± 0.29 ^**a**^	7.96 ± 0.06 ^**a**^	7.96 ± 0.08 ^**a**^
**RBCs count** (* 10^6^ cell/mm^3^)	2.77 ± 0.04 ^**b**^	2.85 ± 0.04 ^**b**^	3.58 ± 0.1 ^**a**^	2.88 ± 0.04 ^**b**^
**MCV (fl.)**	136.74 ± 0.93 ^**a**^	134.04 ± 0.47 ^**ab**^	110.25 ± 1.71 ^**c**^	133.35 ± 0.52 ^**ab**^
**MCH (pg)**	28.31 ± 0.85^**a**^	28.59 ± 0.64 ^**a**^	22.3 ± 0.49 ^**b**^	27.65 ± 0.18 ^**a**^
**MCHC (%)**	20.7 ± 0.09 ^**ab**^	21.33 ± 0.49 ^**a**^	20.22 ± 0.15 ^**b**^	20.73 ± 0.12 ^**ab**^
**WBCs count **(* 10^3^ cell/mm^3^)	11.08 ± 0.31 ^**b**^	11.30 ± 0.24 ^**b**^	14.96 ± 0.39 ^**a**^	11.89 ± 0.32 ^**b**^
**Heterophile (H) %**	29.81 ± 0.71 ^**b**^	29.47 ± 0.51 ^**b**^	41.23 ± 1.5 ^**a**^	31.10 ± 0.33 ^**b**^
**Lymphocyte (L) %**	65.19 ± 0.64 ^**a**^	65.53 ± 0.49 ^**a**^	53.77 ± 0.9 ^**b**^	63.90 ± 0.32 ^**a**^
**H/L**	0.46 ± 0.02 ^**b**^	0.45 ± 0.01 ^**b**^	0.77 ± 0.05^**a**^	0.48 ± 0.008 ^**b**^

^a, b, c^ Means within a row with different superscripts significantly differ (Tukey’s test; *P* < 0.05)

TN (control): Thermoneutral treatment, fed basal diet; TN + PHY: Thermoneutral treatment fed basal diet + 1 kg/ton feed PHY; HS: Heat stress treatment, fed basal diet; HS + PHY: Heat stress treatment, fed basal diet + 1 kg/ton feed PHY.

Hb conc: Hemoglobin concentration; RBCs count: Red blood cells count; MCV: Mean corpuscular volume; MCH: Mean corpuscular hemoglobin; MCHC; Mean corpuscular hemoglobin concentration; WBCs count: White blood cells count; (H/L) ratio: heterophile/ lymphocyte ratio

Data are means ± SEM (standard error of the mean)

Number of sampled birds (n) = 3 birds/replicate (12 birds/ treatment)

### Metabolic parameters

The PHY fortification improved lipid profile where it significantly decreased cholesterol, triglyceride, and VLDL cholesterol levels when compared to the control treatment, while the HS treatment displayed negative impacts on lipid profile where it significantly increased cholesterol, triglyceride, LDL & VLDL cholesterol levels when compared to the TN treatment (*P < 0.05).* Furthermore, the HS + PHY treatment could enhance the ameliorating effects of HS where it showed no significant difference in lipid profile when compared to the TN treatment (*P > 0.05*) as displayed in Table 4. The protein profile displayed in Table 4 shows that the HS treatment significantly reduced total protein, albumin, and globulin but increased the A/G ratio when compared to the TN treatment (*P < 0.05*). The HS + PHY treatment significantly increased total protein and globulin while decreasing the A/G ratio when compared to the HS treatment (*P < 0.05*). The glucose level shown in Table 4 displayed a significant increase in the HS treatment (*P < 0.05*) while the other treatment did not show any change compared to the control one (*P > 0.05*).

**Table 4:** Effect of phytogenic feed additives (PHY) on metabolic parameters (lipid profile, protein profile and glucose level) of thermoneutral, and heat-stressed broiler chickens

**Parameters**	TN	TN + PHY	HS	HS + PHY
**Triglycerides**	50.49 ± 1.01 ^**b**^	37.8 ± 1.88 ^**c**^	60.09 ± 0.6 ^**a**^	51.91 ± 1.84 ^**b**^
**Cholesterol**	95.1 ± 1.71 ^**b**^	85.16 ± 1.08 ^**c**^	120.57 ± 3.77 ^**a**^	92.91 ± 1.28 ^**b**^
**“HDL chol”**	72.56 ± 1.76 ^**b**^	67.59 ± 1.39 ^**b**^	76.27 ± 2.78 ^**a**^	69.91 ± 1.55 ^**b**^
**“LDL chol”**	12.43 ± 0.55 ^**b**^	10.01 ± 0.37 ^**b**^	32.29 ± 1.44 ^**a**^	12.62 ± 0.73 ^**b**^
**“VLDL chol”**	10.1 ± 0.20 ^**b**^	7.56 ± 0.38 ^**c**^	12.02 ± 0.12 ^**a**^	10.38 ± 0.37 ^**b**^
**Total protein**	3.27 ± 0.29 ^**a**^	3.26 ± 0.17 ^**a**^	1.95 ± 0.03 ^**c**^	2.7 ± 0.09 ^**b**^
**Albumin**	1.68 ± 0.14 ^**a**^	1.64 ± 0.08 ^**a**^	1.09 ± 0.02 ^**b**^	1.29 ± 0.08 ^**b**^
**Globulin**	1.59 ± 0.16 ^**a**^	1.62 ± 0.11 ^**a**^	0.86 ± 0.01 ^**b**^	1.41 ± 0.02 ^**a**^
**“A/G ratio”**	1.07 ± 0.05 ^**b**^	1.02 ± 0.05 ^**b**^	1.27 ± 0.03 ^**a**^	0.92 ± 0.05 ^**b**^
**Glucose**	147.27 ± 7.34 ^**b**^	144.24 ± 5.66 ^**b**^	225.11 ± 6.09 ^**a**^	160.48 ± 2.48 ^**b**^

^a, b, c^ Means within a row with different superscripts significantly differ (Tukey’s test; *P* < 0.05)

TN (control): Thermoneutral treatment, fed basal diet; TN + PHY: Thermoneutral treatment fed basal diet + 1 kg/ton feed PHY; HS: Heat stress treatment, fed basal diet; HS + PHY: Heat stress treatment, fed basal diet + 1 kg/ton feed PHY.

HDL chol: High-density lipoprotein cholesterol; LDL chol: Low-density lipoprotein cholesterol; VLDL chol: Very low-density lipoprotein cholesterol; A/G ratio: albumin/ globulin ratio

Data are means ± SEM (standard error of the mean)

Number of sampled birds (n) = 3 birds/replicate (12 birds/ treatment)

### Liver and kidney function biomarkers

Heat stress had a negative impact on liver and kidney function tests, with significant increases in ALT, AST, uric acid, and creatinine compared to the TN treatment (*P < 0.05*), while PHY fortification can improve the negative effects that result from HS. It can reduce the parameters that are negatively affected by HS. Additionally, the treatments TN + PHY and HS + PHY did not display any significant changes in liver and kidney function tests when compared to the TN treatment (*P > 0.05*) as shown in Table 5.

**Table 5:** Effect of phytogenic feed additives (PHY) on liver enzymes (ALT and AST) activities, and kidney function tests (uric acid and creatinine levels) of thermoneutral, and heat-stressed broiler chickens

**Parameters**	TN	TN + PHY	HS	HS + PHY
**ALT (U/l)**	3.99 ± 0.28 ^**b**^	3.74 ± 0.18 ^**b**^	12.61 ± 0.79 ^**a**^	4.33 ± 0.26 ^**b**^
**AST (U/l)**	51.45 ± 2.27 ^**b**^	39.0 ± 1.41 ^**c**^	77.4 ± 2.2 ^**a**^	57.2 ± 1.98 ^**b**^
**Uric acid (mg/dl)**	3.35 ± 0.18 ^**b**^	3.48 ± 0.25 ^**b**^	8.06 ± 0.60 ^**a**^	3.90 ± 0.13 ^**b**^
**Creatinine (mg/dl)**	0.45 ± 0.04 ^**b**^	0.38 ± 0.04 ^**b**^	0.79 ± 0.03 ^**a**^	0.5 ± 0.03 ^**b**^

^a, b, c^ Means within a row with different superscripts significantly differ (Tukey’s test; *P* < 0.05)

TN (control): Thermoneutral treatment, fed basal diet; TN + PHY: Thermoneutral treatment fed basal diet + 1 kg/ton feed PHY; HS: Heat stress treatment, fed basal diet; HS + PHY: Heat stress treatment, fed basal diet + 1 kg/ton feed PHY.

ALT: Alanine aminotransferase ; AST: Aspartate aminotransferase

Data are means ± SEM ( standard error of the mean)

Number of sampled birds (n) = 3 birds/replicate (12 birds/ treatment)

### Oxidative stress biomarkers

**Fig. 5:**
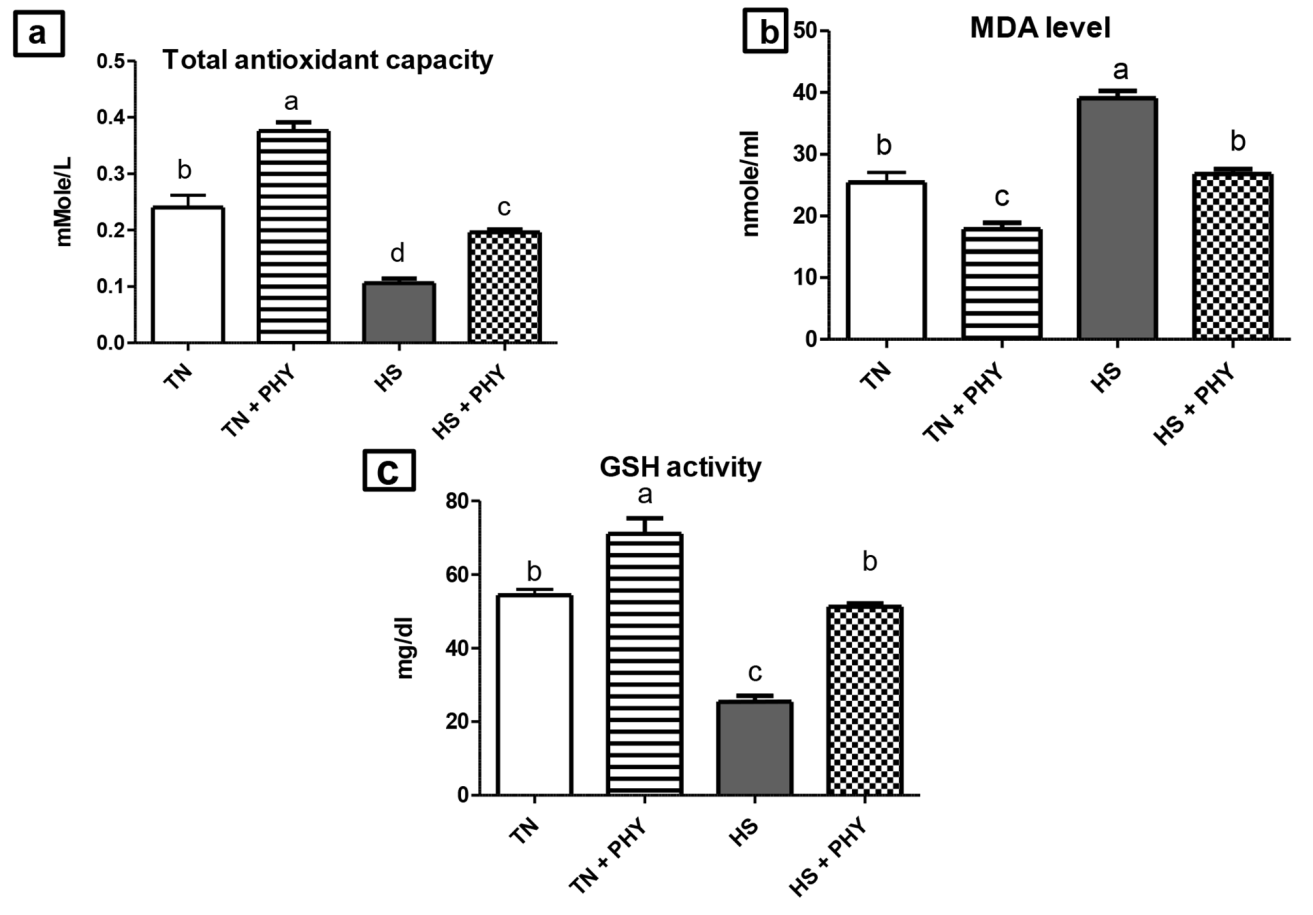
Effect of phytogenic feed additives (PHY) on oxidative stress biomarkers: **(a)** total antioxidant capacity, **(b)** malondialdehyde (MDA) level, and **(c)** reduced glutathione (GSH) activity of heat-stressed broiler chickens; Data are presented as mean ± standard error (*n* = 12). Different alphabets indicate significant differences at *P* < 0.05

The total antioxidant capacity and reduced glutathione activity decreased while MDA rose in the HS treatment compared to the TN treatment. The PHY fortification improved antioxidant activity where it increased TAC, and GSH activity, but lowered MDA level when compared to the TN treatment (*P < 0.05*). The HS + PHY treatment showed a significant increase in TAC, and GSH but, a decrease in MDA level in comparison to the HS treatment (*P < 0.05*) as shown in Fig. 5.

### Gene expression analysis

#### Heat stress-responsive gene (*HSP70*)

**Fig. 6:**
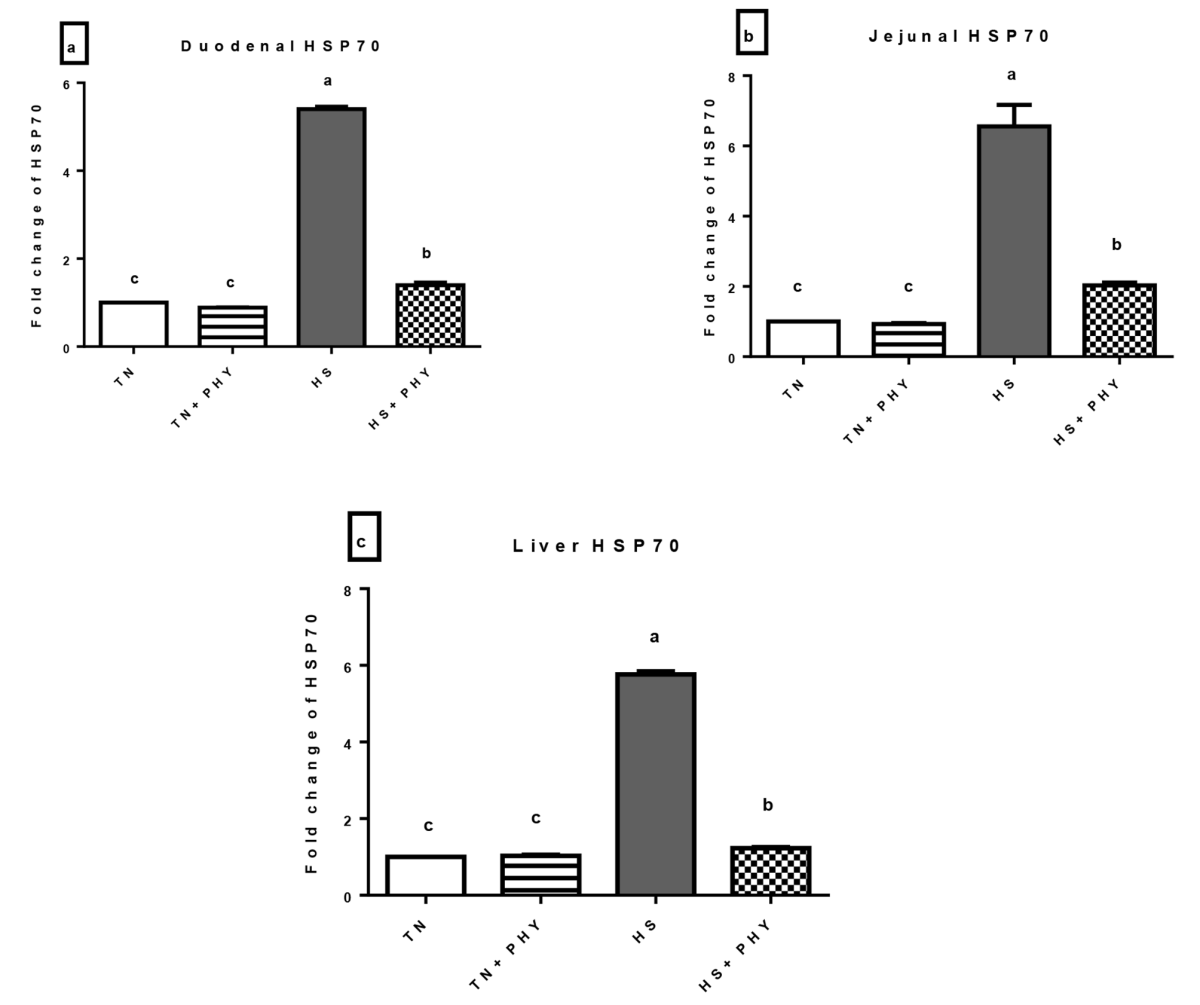
Effect of phytogenic feed additives (PHY) on the relative expression level of **(a)** duodenal **(b)** jejunal and **(c)** liver *HSP70* gene of heat stressed broiler chickens; Data are presented as mean ± standard error (*n* = 12). Different alphabets indicate significant differences at *P* < 0.05

In the present study, the *HSP70* showed the highest significant up-regulation in the HS treatment followed by the HS + PHY in the liver, duodenum, and jejunum (*P < 0.05*). Meanwhile, there was no significant difference between the TN treatment and the TN + PHY treatment (*P > 0.05*) (Fig. 6).

#### Gut health biomarker (*I-FABP2*)

**Fig. 7:**
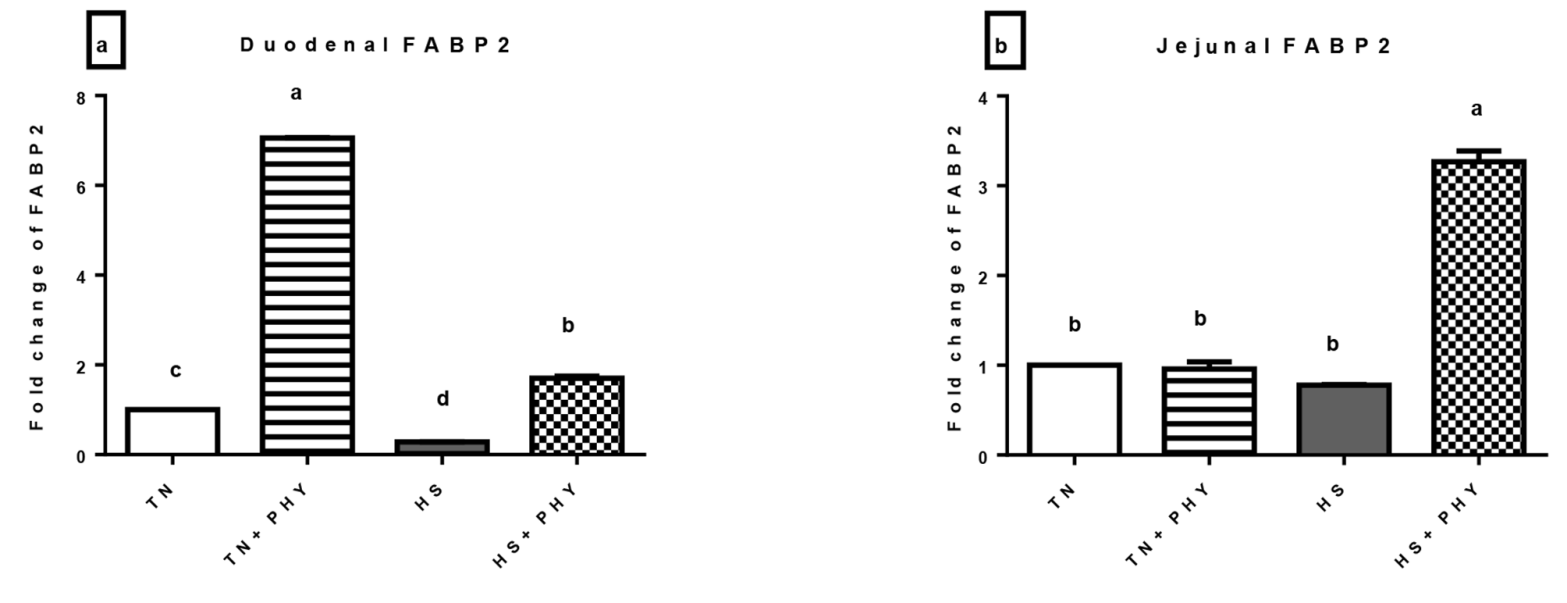
Effect of phytogenic feed additives (PHY) on the relative expression level of **(a)** duodenal **(b)** jejunal and **(c)** liver *FABP2* gene of heat stressed broiler chickens; Data are presented as mean ± standard error (*n* = 12). Different alphabets indicate significant differences at *P* < 0.05

The *I-FABP2* expression pattern in the duodenum showed significant upregulation in the TN + PHY and HS + PHY treatments. Meanwhile, it was downregulated in the HS treatment (*P < 0.05*). Regarding its expression in the jejunum, it showed no significant difference between treatments except for the HS + PHY treatment which showed significant upregulation (*P < 0.05*) (Fig. 7),

#### Immunity-related genes (*TLR4* and *IL10*)

**Fig. 8:**
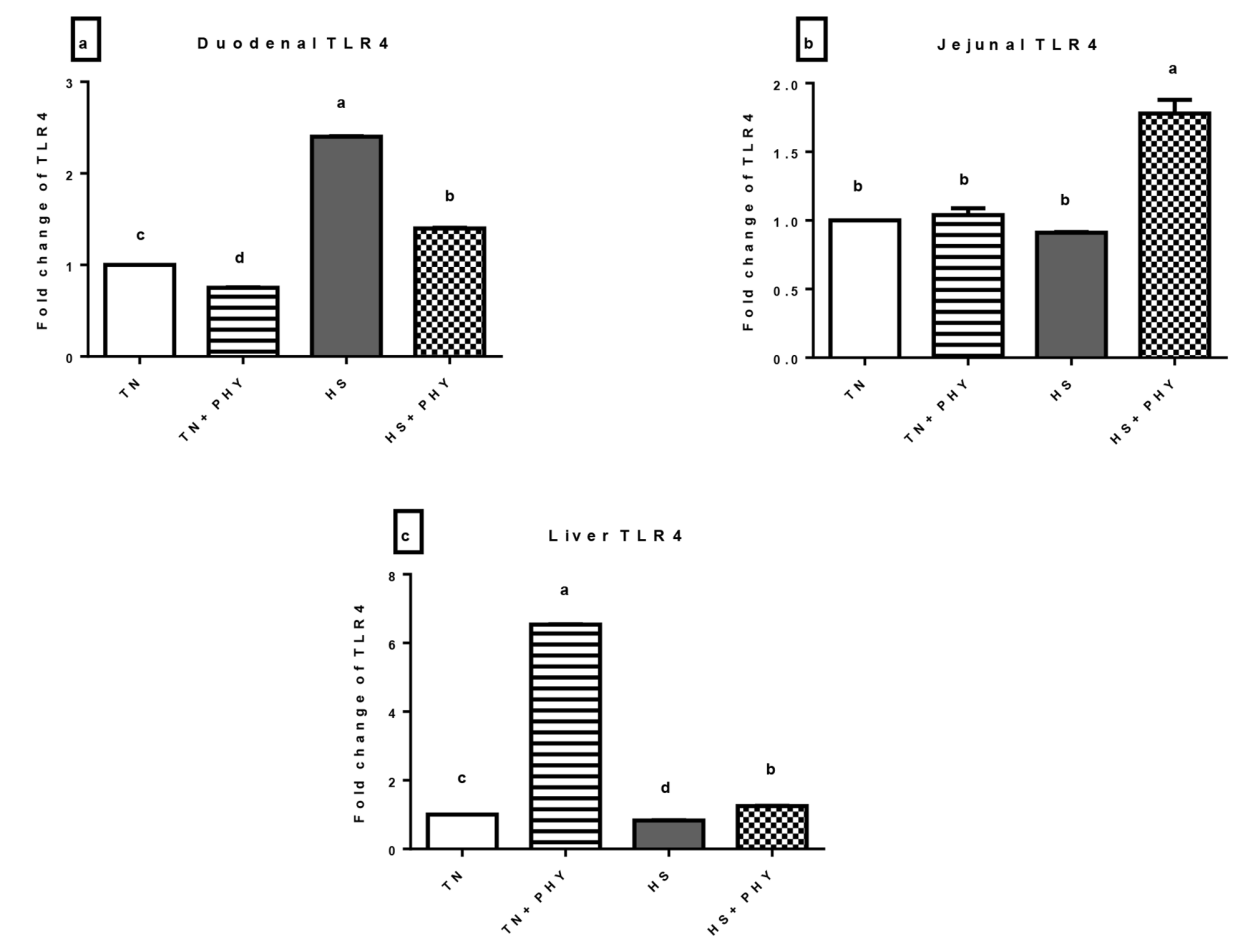
Effect of phytogenic feed additives (PHY) on the relative expression level of **(a)** duodenal **(b)** jejunal and **(c)** liver *TLR4* gene of heat stressed broiler chickens; Data are presented as mean ± standard error (*n* = 12). Different alphabets indicate significant differences at *P* < 0.05

As shown in Fig. 8, the *TLR4* gene in the liver showed significant upregulation in the TN + PHY and HS + PHY treatments, while it was downregulated in the HS treatment (*P < 0.05*). Duodenal *TLR4* showed significant upregulation in the HS and HS + PHY treatments, while it was downregulated in the TN + PHY treatment (*P < 0.05*). Jejunal *TLR4* showed significant upregulation in the HS + PHY treatment while the rest of the treatments showed no significant difference from the TN treatment (*P > 0.05*).

**Fig. 9:**
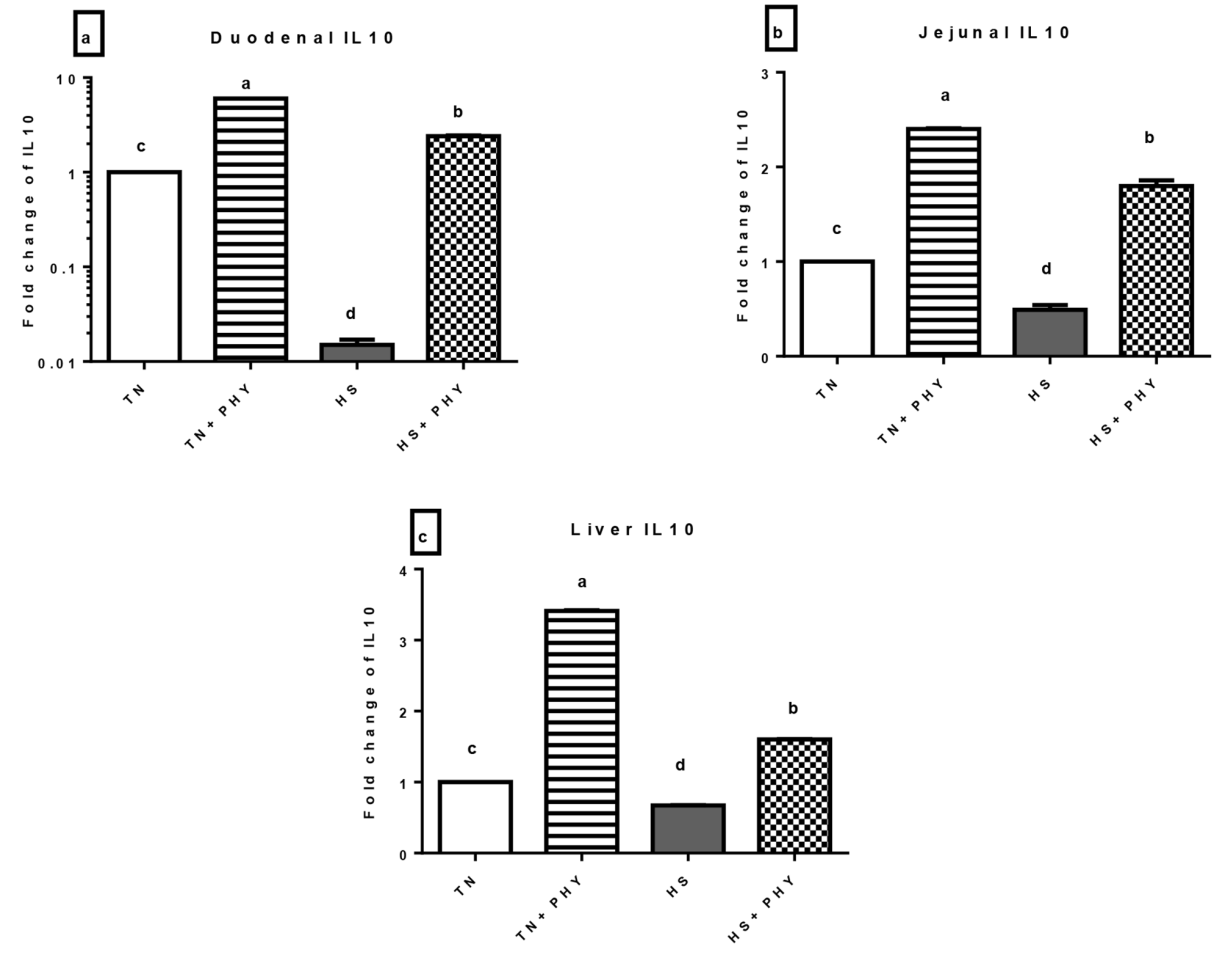
Effect of phytogenic feed additives (PHY) on the relative expression level of **(a)** duodenal **(b)** jejunal and **(c)** liver *IL10* gene of heat-stressed broiler chickens; Data are presented as mean ± standard error (*n* = 12). Different alphabets indicate significant differences at *P* < 0.05

The *IL-10* showed marked-up regulation in hepatic, duodenal, and jejunal tissues for the TN + PHY treatment, and in the HS + PHY treatment (*P < 0.05*). Meanwhile, the HS treatment showed significant downregulation in the hepatic, duodenal, and jejunal tissue (*P < 0.05*) (Fig. 9).

#### Nutrient-sensitive pathway (*mTOR*)

Regarding the expression level of the *mTOR* gene, it showed a marked significant up-regulation in the hepatic and duodenal tissue of the TN + PHY treatment, and the duodenum and jejunum of the HS + PHY treatment (*P < 0.05*).

**Fig. 10:**
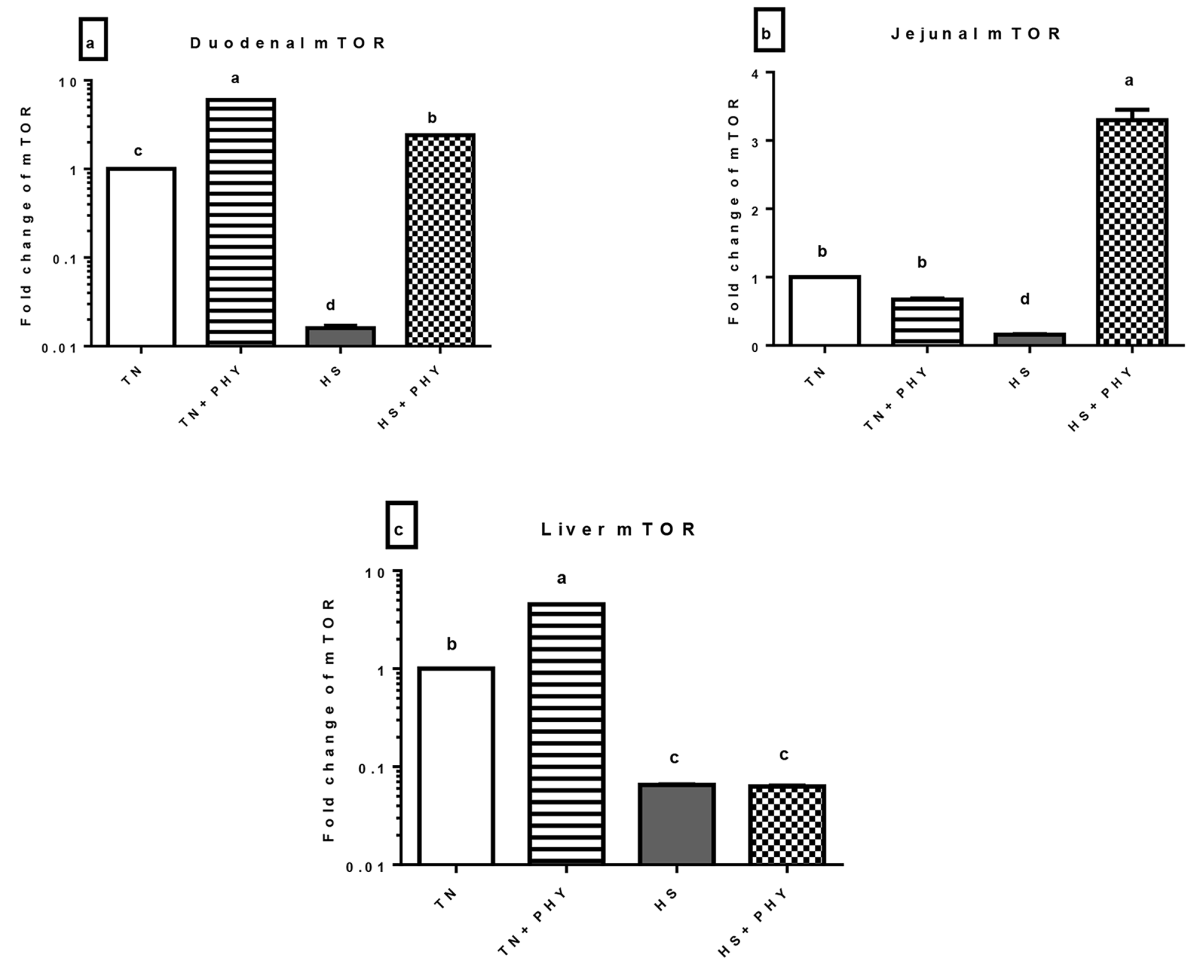
Effect of phytogenic feed additives (PHY) on the relative expression level of **(a)** duodenal **(b)** jejunal and **(c)** liver *mTOR* gene of heat-stressed broiler chickens; Data are presented as mean ± standard error (*n* = 12). Different alphabets indicate significant differences at *P* < 0.05

The *mTOR* was observed to be downregulated in the HS and HS + PHY treatments for the liver. Also, the jejunum of HS and TN + PHY treatments showed downregulation in *mTOR*, while in the duodenum; only the HS treatment showed significant downregulation (*P < 0.05*) (Fig. 10).

## Discussion

Global research efforts have increased to identify alternative supplements in response to the “raised without antibiotics” demand and the prohibition on their subtherapeutic use as feed additives [64]. Animal performance is enhanced by the use of phytogenic or phytobiotic feed additives, which are made from plants, herbs, and spices [65]. The PHY additives positive effects on growth, immune system, and stress-relieving response have made them extremely successful, even though the underlying mechanisms are not fully understood. The current study aimed to ascertain the impact and mode of action of a new polyherbal formulation of *Terminalia bellirica* and *Andrographis paniculata* on the broilers raised under HS conditions.

In earlier studies, the phytochemical analysis of *Andrographis paniculata* revealed the presence of 32 bioactive compounds including diterpene lactones (deoxyandrographolide, andrographolide, neoandrographolide, and 14-deoxy-11, 12-didehydroandrographolide), diterpene glucoside, 30 flavonoids, 5 noriridoids, 7-ent-labdane diterpenoids, 2 quinic acid derivatives, 55-ent-labdane diterpenoids, 4 xanthones, 8 quinic acids, and andrographidoids [66–68]. Nonetheless, it has been discovered that the primary component, andrographolide, is in charge of its essential medicinal qualities [69]. Meanwhile, *T. bellirica* contains several major phytoconstituents, including triterpenoids, flavone, tannins, poly phenols, phenyllemblin, galloyl glucose, ethyl gallate, ellagic acid, bellericanin, chebulaginic acid, gallic acid, corilagin, ellargic acid, termilignan, β-sitosterol.,and thannilignan [70, 71].

### Effects of PHY on behavioral parameters

Behavioral parameters could be regarded as a valuable tool for assessing the welfare state of the broiler [72]. It is well known that when birds are exposed to a stressful stimulus like thermal stress their behavior changes to adapt to the new situation [73]. The presence of significant negative effects of HS on broiler behavior and physiology was observed in our results. High ambient temperature in birds caused heat-associated behaviors including decreased foraging and feeding behavior and increased drinking behavior, these results in concurrent with [74] who stated that feeding and drinking behavior can be an adaptive mechanism in the face of thermal stress. Previous research has shown that birds exposed to HS reduce their feeding activities to control their metabolic energy production [75] and increase water intake to cool their bodies to fulfill the immediate demands of evaporative cooling from respiratory surfaces during HS [76]. Since dehydration generally prevents the evaporative reaction to heat exposure, therefore maintaining water balance aids in heat dissipation and consequently controls the body temperature [77].

Our findings demonstrated that feeding PHY feed additives can significantly minimize the deleterious effects of chronic HS on the physiological and behavioral alterations of broilers. Using dietary polyherbal compounds containing *T. Bellirica* and *A. paniculata* in the current study demonstrated a positive effect on foraging, feeding, and drinking behavior of broilers. These changes could be attributed to the improved body heat metabolism and appetite [32, 78], as *T. Bellirica* and *A. paniculata* contain numerous bioactive metabolites including alkaloids, flavonoids, tannins, and phenols [31, 37], that have anti-inflammatory, antibacterial and antipyretic effects [33, 38].

When the environmental temperature increases above the thermoneutral zone, it was found that, birds engage in more resting in conjugation with less time walking/standing as observed in previous research [79]. Lying down could encourage a decrease in the production of metabolic heat and aid the bird in achieving thermal balance [74]. thereby, coping with HS or reducing the negative consequences of HS [79]. Birds’ walking activities were significantly increased by feeding the dietary PHY additives while the resting activities were decreased. This could be attributed to the improvement of bone health of polyherbal-fed birds and therefore their locomotor activities [80, 81]. Because *T. bellirica* contains a lot of calcium (ca.) and magnesium (Mg), so it is one of the greatest herbs for bone healing [34]. Additionally, *A. paniculata* has been demonstrated to be a good source of potassium (K), Ca, Mg, ferrous (Fe), Aluminum (Al), and sodium (Na), all of which are important for health and bone integrity [82]. The comfort behavior patterns used as a stress indicator in broilers decreased under HS conditions [83]. These behavioral changes could be attributed to the oxidative stress caused by chronic HS [84, 85], and improvement of comfort behavior via PHY additives is related to their antioxidant properties [30, 37].

### Effects of PHY on hematological parameters

Heat stress can affect the chicken’s hematological status and disrupt their immune system. Blood components in chickens are highly sensitive to environmental changes, making them a valuable indicator of physiological changes [86]. According to the study, the HS treatment had the highest RBCs count and the lowest MCV and MCH values. Additionally, their WBCs count increased, primarily due to an increase in heterophile percentage, while their lymphocyte percentage decreased. These changes led to a significant increase in the H/L ratio as reported by [87, 88]. Exposure of chicken to stressors such as temperature higher than thermal comfort zone led to activation of the hypothalamus-pituitary-adrenocortical (HPA) axis resulting in a rapid increase in circulatory corticosterone levels which in turn may increase H/L ratio, retarding erythrophagocytosis, and stimulating erythropoiesis [9, 89]. Thermal stress can cause a decrease in lymphocyte count, immunoglobulin, antibody response, and macrophage phagocytic activity in broiler chickens [90]. The main active ingredient of *andrographis paniculata* extract, andrographolide, has been shown to have strong anti-stress properties and helpful in the treatment of behavioral disturbances by modulating biological processes that control corticosterone and cytokine homeostasis [91]. The hypothalamic-pituitary-adrenal axis hyperactivity has been significantly regulated by the gallic acid extract from *T. bellirica* fruits by lowering serum corticosterone and acetylcholinesterase levels in chronic mild stress-induced depression-like activity in mice model [92].

### Effects of PHY on metabolic parameters

Broiler health issues can be identified by analyzing blood biochemistry. The HS treatment showed increased levels of triglycerides, cholesterol, LDL, VLDL, and glucose, while total proteins and globulins were reduced. Generally, exposing birds to adverse environmental temperatures results in a decline in thyroid activity and protein contents while increasing protein catabolism, metabolic acidosis, and anaerobic glycolysis., which leads to an increase in muscular fat deposition [93]. Blood glucose levels increase due to corticosteroid-stimulated gluconeogenesis, also, changes in corticosterone concentration affect body composition, meat quality, and protein and lipid metabolism [94].

The PHY fortification can significantly reduce triglycerides, cholesterol, LDL, and VLDL levels compared to other treatments. It can also ameliorate the adverse effects of HS by improving lipid, glucose, and protein profiles. It can affect the sympathetic-adrenal axis, thereby reducing corticosterone synthesis and increasing cholesterol clearance endogenously, this leads to a decrease in triglycerides and cholesterol levels in the body [95, 96]. Wiono et al. [87] found that Phytocee™, a PHY feed additive, lowered lipid parameters and increased total protein levels in heat-stressed broilers, which is consistent with previous studies.

It has been reported that rats with hyperlipidemia caused by Porphyromonas gingivalis [97] and also those in high-fat emulsion (75% yolk)-diet [98] had lower total cholesterol, LDL-C, and triglyceride levels after receiving andrographolide. Gallic acid (20 mg/kg) from *T. bellirica* fruit restored serum total cholesterol, triglycerides, LDL cholesterol, urea, uric acid, creatinine, and increased plasma insulin, C-peptide, and glucose tolerance in STZ-induced diabetic rats after 28 days of oral administration [99]. In addition, gallic acid is known to increase the ratio of Bacteroidetes/Firmicutes and gut bacteria that produce SCFAs (short chain fatty acids) to maintain gut health and lower blood triglyceride and intestinal fat digestibility [100]. Another possible mechanisms for hypolipidemic effect of *T. bellirica* may be attributed to the gallic acid activation of *AMPKα*, which inactivates the *ACC-PPARα* axis signaling, and may be responsible for the liver lipid-lowering effects of *T. bellirica* [101]. Additionally, corilagin may be involved, as it has been shown to downregulate the expression of mRNA related to fatty acid synthesis (*FASN*, ACC1, SREBP-1c) and upregulate the expression of genes related to fatty acid oxidation (*PPARα*, *CPT1α*,* ACOX1*) [102]. Compared to simvastatin, *T. bellirica* appeared to elicit a larger biological response inferred from serum markers (ALT and AST) and hepatic inflammatory cytokines (*IL-1β*,* IL-6*, and *TNF-α*) [103]. Flees et al. [65] also reported that PHY additives can modulate avian lipid metabolism via reducing hepatic lipogensis and enhancing the ß-oxidation. Morever, ethanolic *T. bellirica* extract was reported to slow down the onset of NAFLD (Non-alcoholic fatty liver disease) by lowering the glycerol 3-phosphate (G3P) content since G3P is the synthetic substrate of triglycerides and fatty acids [104].

Recently, it has been shown that Andrographolide can regulate hepatic apelin expression levels in rats and the increment in apelin gene expression was associated with decreased serum glucose levels [105]. *A. paniculata* showed its potential to increase the expression of the glucose transporter type 4 (*GLUT4*), increase the number of pancreatic beta cells, which in turn increases insulin secretion, and promote the regeneration of pancreatic beta cells [106]. *T. bellirica* hypoglycemic activity could be attributed to the synergistic action between more than one compound, like the presence of polyphenolic compounds that suppress elevated plasma glucose levels. Tannins have been shown to have insulin-like glucose transport stimulatory properties. Furthermore, gallotannins like pentagalloyl glucose are more potent and effective at binding to the insulin receptor (IR), activating the IR, and inducing glucose transport [107]. In alloxan diabetic rats both aqueous and ethylacetate *T. bellirica* extracts showed a restorative effect on serum biomarkers like glucose, creatinine, total protein, total cholesterol, LDL, HDL, triglyceride, urea, and uric acid [108]. *T. bellirica* notably decreased blood glucose levels in HFD (high-fat diet)-STZ-induced diabetes in rats [109].

### Effects of PHY on oxidative stress biomarkers

Heat stress is a well-known environmental issue that can increase oxidative stress levels in the body’s cells [9]. The HS in broiler chickens increases cellular reactive oxygen species (ROS) levels, which impairs the effectiveness of the antioxidant system. This, in turn, reduces enzymatic antioxidant activities like GPx, SOD, and CAT, as well as total antioxidant capacity. Meanwhile, the levels of malondialdehyde (MDA) rise [5, 110]. The current results demonstrated that HS negatively disturbed the redox balance with increasing MDA levels and decreasing GSH and total antioxidant concentration as reported by [111]. Our results showed that the PHY additive boosted the antioxidant activity, increasing TAC and GSH activity while decreasing MDA levels. High environmental temperatures were reported to induce oxidative stress, and hepatic as well as renal injury in animals [112]. Gupta et al. [30]; Owoade et al. [37] related the antioxidative effects of *T. Bellirica* and *A. paniculata* to its high content of polyphenols, which reduce the impact of HS on the physiological responses of broiler chickens [113]. Many possible mechanisms exist to account for andrographolide’s antioxidant activity. These mechanisms can be direct [114] or indirect [115]. Andrographolide can inhibit relevant ROS-producing enzymes or protect mitochondria to prevent the generation of free radicals [116]. It may also stimulate enzymatic (SOD, CAT, GST, GSH, and GPx) or non-enzymatic antioxidants, primarily through activating the Nrf2 signaling pathway. *T.bellirica* fruit contains a variety of polyphenols, including gallic acid and ellagic acid [117]. According to Middha et al. [118], gallic acid and ellagic acid have free radical scavenging activity and inhibit lipid peroxidation. Also, they have been demonstrated to inhibit lipoxygenase activity and lipoxygenase-mediated LDL lipid peroxidation [119]. These findings indicate that *T.bellirica* may have an antioxidant impact on LDL oxidation by inhibiting both the radical reaction by free radicals and the non-radical reaction by peroxidative enzymes [120]. In addition, T. bellirica’s bioactive compounds enhanced the antioxidant response by activating *Nrf2* (nuclear factor erythroid-2 related factor 2), *PI3K/Akt*, and *AMPK* transcriptionally [121].

### Effects of PHY on liver and kidney functions

To monitor the liver’s health, the levels of ALT and AST in the serum of the broiler are measured. Our study have shown that the levels of ALT and AST were higher in the treatment exposed to HS, while they remained unchanged in the treatments given PHY feed additives compared to the TN treatment. Furthermore, for kidney health evaluation, the uric acid and creatinine values in the HS treatment were observed to be higher than all other treatments. This increase in values could be due to a series of reactions in the nervous and endocrine systems, leading to the release of corticotropin hormone [122]. Fortunately, a study by [87] demonstrated that Phytocee™ effectively controlled HS and had no harmful effects on liver and kidney functions. Rats treated with *T. bellirica* fruit extract and its ellagic acid derivative showed hepatoprotective effects against liver damage induced by longterm use of diclofenac [123]. Also, pre-treatment with 100–200 mg/kg of *T. bellirica* leaf methanolic extract markedly and dose-dependently showed hepatoprotective potential against d-galactosamine (D-GalN)-induced liver injury in rats [70]. *T. bellirica* fruit ethyl acetate extract and ellagic acid had hepatoprotective effects in aceclofenac-induced hepatotoxicity in rats [124]. These hepatoprotective effects were represented in decreased levels of serum markers including ALT, AST, GGT, bilirubin, and ALP [70, 124]. *A. paniculata* has been reported to have an ameliorative effect for methotrexate (MTX) induced nephrotoxicity in rats and restored kidney markers including BUN, urea, creatinine, and uric acid [125]. *T. bellirica* showed a marked restorative effect on kidney function defined by significant improvement of serum urea, uric acid, and creatinine levels in either alloxan or STZ-induced diabetes in rats [99, 108]. Moreover, *T. bellirica* proved its nephroprotective effect by enhancing the kidney’s antioxidant status [121]. Also, *T.* bellirica was reported as a natural substitute for lowering serum uric acid due to its ability to inhibit xanthine oxidase, which has enhanced both the estimated glomerular filtration rate and serum creatinine [126].

### Effects of PHY on gene expression

It’s well identified that HS results in the decrease of protein synthesis except for the highly conserved proteins known as heat shock proteins (*HSPs*) [127]. The *HSPs* are a group of molecular chaperones that direct the proper folding of newly synthesized proteins and refolding of the misfoldedones. As a surviving mechanism, The *HSPs* are found to be upregulated in response to exposure of cells to stressful conditions like; high temperatures or oxidative stress [128, 129]. In the present study, as expected, *HSP70* was significantly upregulated in the liver, duodenum, and jejunum of the HS treatment in comparison to the other treatments as reported by [130, 131]. Meanwhile, the PHY fortification reduced the *HSP70* expression in the HS treatment. Similarly, PHY feed additives (comfort ™) decreased *HSP70* in the hypothalamus of chronic HS broilers [132]. Comparably, Dietary resveratrol significantly reduced *HSP70*, *HSP90*, and *NF-κB* expression in the gut of HS chickens after 2 weeks of supplementation [133]. The possible mechanism of action for *A. paniculata* can be attributed to Andrographolide which in response to elevated ROS, stimulates heat shock factor 1 (*HSF1*) which is the key regulator for HSR (Heat shock response) and upregulates the number of genes like inducible protein chaperons as HSP70 [134]. Meanwhile, among *T. bellirica* phytochemical constituents; ellagic acid was reported to mitigate the effects of different stressors via the downregulation of *HSP70* [135, 136].

Heat stress is thought to primarily target the intestine [4]. Consequently, it is essential to preserve the gut barrier’s proper functioning for the body’s general health and balance as well as for preserving its ability to defend against environmental antigens. A collection of fatty acid transporters and binding proteins facilitates the intestine’s absorption of the majority of dietary fats [137]. *FABP2*; intestinal *FABP*; was found to be abundantly expressed in the enterocytes and had a crucial role in the gut barrier integrity [138].

To delineate the effect of PHY feed additives on gut health; *FABP2* expression levels were investigated in the duodenum and jejunum. Our result showed that *FABP2* was significantly downregulated in duodenum of the HS treatment while jejunum was not affected. Meanwhile its up regulated in both the duodenum and jejunum of the HS + PHY treatment in addition; the duodenum of the TN + PHY treatment. The HS alters the integrity of the intestine, which increases permeability in the gut and thus facilitates pathogen invasion, and impairment of nutrient absorption, resulting in a lowering of the animal performance. Numerous investigations have documented a reduction in the intestinal expression of the *FABP* gene in chickens, regardless of the duration and degree of stress exposure [139, 140]. The decrease in the levels of the FABP gene during HS could be attributed to structural damage and epithelium loss in the intestine [141]. *I-FABP* is thought to be a biomarker of intestinal barrier breakdown in various mammals, including humans [142]. Chen et al. [143] reported that broilers with compromised gut barriers had lower levels of *FABP-2* expression. The possible restorative mechanism is due to maintaining the health and integrity of the intercellular structure of the enterocytes via the antioxidant effect of *A.paniculata* and *T. bellirica* through reducing ROS and activating the *NRF2* pathway [121], also it has been reported that dietary andrographolide upregulated the occludin, *ZO-1* (zonula occludens-1) and *ZO-2* (zonula occludens-1) mRNA levels which stabilize intercellular structural integrity of the intestine in *Monopterus albus* [144], in addition, andrographolide has proven to have gut microbiome modulating effect which is critical for the intestinal health [144, 145]. Ethyl acetate extract from *Terminalia bellirica* (TBEA) preserved intestinal homeostasis by controlling the composition of the intestinal flora, lowering the concentration of inflammatory cytokines as well as ROS in mice with ulcerative colitis [146]. *T. bellirica* extract showed its ability to prevent *S. Typhimurium* infection in mice by enhancing the intestinal physical and immunological barriers and restoring the gut microbiota [147]. According to Li et al. [148], gallic acid may be able to mitigate the colitis caused by dextran sulfate sodium in rats by modifying the composition of the microbiome. Furthermore, by boosting the overall level of probiotics like Bifidobacterium, ellagic acid may mitigate the dysbiosis of the gut microbiota brought on by alcohol [149]. Similarly, *Spirulina platensis* supplementation to broilers’ diet was reported to improve the gut health barrier by increasing the expression of FABP2 in the jejunum and enhancing the gut microbiota [62].

Pro- and anti-inflammatory cytokines are secreted by distinct immune cells under varying stress conditions, and they are essential in determining an organism’s immune status [150]. Interleukin-10 (*IL-10*) is one of the most significant cytokines associated with numerous pathophysiological circumstances, where it constrains the production of pro-inflammatory mediators [5]. In the current study, *IL-10* showed significant upregulation in the investigated tissues of all PHY-supplemented treatments either in TN or HS conditions. The HS-induced oxidative stress was reported to raise the levels of inflammatory mediators (*TNF-α*, *IL-2*, and *IFN-γ*) and lower the expression of anti-inflammatory mediators (*IL-10*, for example), which in turn leads to immunological dysfunction [21, 146].

The PHY feed additives successfully raised the *IL-10* gene’s expression level, indicating that HS-induced inflammation in the broiler was being reduced. Since stress triggers inflammatory reactions, the upregulation of the anti-inflammatory cytokine *IL-10* protects cells from the damaging effects of oxidative stress through the production of pro-inflammatory cytokines, innate immunity aids in the removal of pathogens and provides cellular protection; however, reduced levels of these cytokines may encourage bacterial colonization [151, 152]. Andrographolide was reported to increase the anti-inflammatory factor *IL-10* levels in the peritoneal cavity fluid of mice with cecal ligation and puncture (CLP)-induced sepsis [153]. Also, the aqueous leaf extract of *A. paniculata* showed a pronounced effect in the restoration of *IL-10* levels in the intestine and kidney of MTX(Methotrexate) induced toxicity rat model [125]. Gallic acid, as one of the main constituents in *T. Bellirica*, was reported to attenuate the symptoms of collagen-induced arthritis (CIA) via upregulating the anti-inflammatory cytokines including *IL-10* in mouse models [154]. Moreover, gallic acid increased the expression levels of *IL-10* in mice models with atopic dermatitis-like skin inflammation [155].

*TLR4* is identified as the primary lipopolysaccharide (LPS) recognition receptor. *TLR4* is essential for starting the body’s early defense against invasive pathogens and the inflammatory reaction [156, 157]. In the present study, our results revealed that *TLR4* was significantly up-regulated in the duodenum of HS, and HS + PHY; jejunum of HS + PHY treatment, and liver of the TN + PHY treatment. It was reported that heat-stressed broilers either had increased levels of *TLR4* in the duodenum [158] or the jejunum and ileum [130]. According to [159], Andrographolide controls *TLR4* activity and modulates the adaptive immune response. Extract from *Terminalia bellirica* was reported to diminish LPS-induced inflammatory process and oxidative stress in mice [121]. Many studies have reported the inhibitory effects of *T. bellirica* and *A. paniculata* for the TLR4 signaling pathways [103, 160], however, *TLR4* expression-associated diseases are associated with prolonged stimulation for *TLR4* as well as disrupted trafficking via the endo-lysosomal compartment [161]. Meanwhile, our findings can be explained on the basis that PHY feed additives have ameliorated the effect of *TLR4* activation via increasing the anti-inflammatory cytokine (*IL10*) as well as reduced oxidative stress in supplemented treatment**s.**

Serine/threonine kinase *mTOR* regulates many cellular processes in response to growth factors, nutrients, intracellular ATP, and stressors [162–164]. In poultry, *mTOR* promotes protein synthesis and encourages muscle fiber growth and hypertrophy [165]. Our experimental conditions showed that HS downregulated the *mTOR* expression in different organs, while phytonutrient-supplemented treatments showed a significant increase in the expression except for the liver in HS supplemented treatment. In vitro, Andrographolide ameliorated the protein aggregation-induced cellular toxicity via upregulation of *mTORC1* which in turn activated *HSF1* and *Nrf2* [134]. Under TN conditions, using Superliv concentrate premix which contains *Andrographis paniculata* as one of its ingredients resulted in the upregulation of *mTOR* expression, and total protein in broiler muscles [65]. He et al. [21] revealed that heat exposure inhibited *mTOR* activity in chickens. Likewise, it was noted that heat-stressed broilers fed diets nourished with noni, a plant source that contains a range of phytochemicals and antioxidants, had a decrease in *mTOR* mRNA levels in the liver [166]. By lessening the load of protein synthesis during HS, *mTOR* reduction in the liver may serve as a protective mechanism [167]. This could explain our findings that combined phytonutrients (*T. Bellirica* and *A. paniculata*) have decreased the expression levels of *mTOR* in the liver to alleviate the HS–induced oxidative stress and reduce the synthesis of inflammatory cytokines.

There are certain limitations even though our study demonstrated that giving broilers PHY feed additives supplementation has mitigated the negative effects of HS referring to that; little data are available about the effects of *Terminalia bellirica* and *Andrographis paniculata* in poultry. The limitations can be concluded as the following: the phytochemical analysis for the “Herb-ALL™ COOL” is not performed in this study, however, many studies have previously described the phytochemical composition of *Terminalia bellirica* and *Andrographis paniculata* [66–68]. In this study, the recommended inclusion rate by manufacturer for “Herb-ALL™ COOL” has been used, and further studies are needed to test the effects of different doses on the broiler performance. To better understand the effects of *Terminalia bellirica and Andrographis paniculata* on the gut microbiota, meat quality, in laying hens, and in other stressful situations; more research should be conducted. Comparative analysis for changes of different biological biomarkers between blood and different tissue should be conducted. Molecular-based studies are required to confirm the mechanism of action of these plants in the avian species.

## Conclusion

It is concluded that the global poultry industry faces a considerable socioeconomic challenge in the form of HS. This is because HS negatively impacts bird behavior, as well as various physiological and biochemical parameters. These effects result in oxidative stress and a decline in bird productivity. *Terminalia bellirica* and *Andrographis paniculata* supplements have the potential to alleviate the HS in birds. The positive outcomes observed may be attributed to the enrichment of antioxidants in the birds through the addition of PHY feed additives. This fortification seemed to alleviate HS in the animals, resulting in enhancements in their behavior, hematological and biochemical parameters, oxidative stress levels, immune response, and intestinal health. These improvements were evidenced by the regulation of genes associated with heat response, nutrient sensing, and immune function. Furthermore, there was a significant improvement observed in the marker of intestinal health, specifically *I-FABP2*.

## Data Availability

No datasets were generated or analysed during the current study.
